# Therapeutic Drug-Drug Interactions (DDIs) Causing QT Prolongation in Patients With Cancer: A Systematic Review and Meta-Analysis

**DOI:** 10.7759/cureus.82770

**Published:** 2025-04-22

**Authors:** Saravana Kumar Ramasubbu, Archana Mishra, Soumitra Mandal, Chandrahasan K, Prakash B M

**Affiliations:** 1 Department of Pharmacology, Andaman and Nicobar Islands Institute of Medical Sciences, Sri Vijaya Puram, IND; 2 Department of Pharmacology, All India Institute of Medical Sciences, Bhubaneswar, Bhubaneswar, IND; 3 Department of Pharmacology, All India Institute of Medical Sciences, Kalyani, Saguna, IND; 4 Independent Practice, Pharmacology, Mysore, IND

**Keywords:** adverse drug reactions, cancer patients, drug-drug interactions, prevalence, qt prolongation

## Abstract

Therapeutic drug-drug interactions (DDIs) have the potential to harm the patient by causing serious and unwanted side effects. Patients suffering from cancer are exposed to numerous drugs, such as therapeutic drugs and adjuvant treatments for the comorbidities and the side effects of chemotherapy. The concomitant use of anticancer and adjuvant drugs makes such patients prone to QT prolongation, which could precipitate torsades de pointes or TdP. The current review aims to summarize the prevalence of therapeutic DDIs causing QT prolongation in these patients. A thorough literature search was performed using databases such as PubMed, Google Scholar, and Research Gate. Medical Subject Headings (MeSH) and alternate search terminologies such as “QT prolongation”, “Drug interactions”, and “Cancer” were used to identify all the relevant articles published in English to date. The data from all such published articles available on the day of the collection were considered and fed into the meta package of R software for measuring the outcomes. The literature search yielded seven unique and relevant articles, which were included in our study. In these studies, therapeutic DDIs causing QT prolongation were found to occur in 3558 out of 8013 patients undergoing evaluation. Their prevalence in patients with cancer was estimated to be 22% with a 95% CI between 4% and 63%. The patient characteristics, such as age, comorbid diseases, drugs for supportive care, and polypharmacy, were identified as risk factors associated with potential DDIs. Our data concluded that 22% of the patients administered anticancer drugs were exposed to concomitant drugs leading to DDIs, which prolonged the QT interval. Thus, the implementation of vigilant measures and precautionary safety interventions becomes vital to forestall QT prolongation and any other adverse cardiac events in this group of patients.

## Introduction and background

Introduction

The possibility of therapeutic drug-drug interactions (DDIs) and their aftereffects is a holy grail in clinical practice, and occurs when two or more drugs are administered concomitantly and one drug influences the effect of the other, leading to decreased efficacy and/or increased side effects [1,2]. DDIs can harm the patient by causing serious unwanted side effects [3]. Patients with cancer are exposed to numerous drugs, which include drugs to treat the disease, reduce associated pain, and treat comorbid illnesses (diabetes, hyperlipidemias, and cardiac diseases, etc.), along with adjuvant drugs to combat the adverse effects due to cancer chemotherapy. Exposure to multiple drugs makes these patients vulnerable to potentially serious DDIs, and it can get worse if there is a comorbid illness (renal failure, liver failure, and heart failure) [4].

The most notable aspect of cardiac rhythm disorders brought on by anticancer medications and supportive care therapy is the prolonged QT interval, which can eventually result in ventricular arrhythmias, especially when these medications are used concurrently. On the electrocardiography (ECG) rhythm strip, the interval between the initiation of the QRS complex and the end of the T wave is measured as the QT interval. It demonstrates the phases of ventricular depolarization and subsequent repolarization. The rare and deadly polymorphic ventricular tachycardia known as torsades de pointes (TdP) is frequently represented by twisting of the points on an ECG [5,6].

Arsenic trioxide (AsO3), used in the treatment of acute promyelocytic leukemia (APL), when taken at therapeutic doses, may prolong the QT interval and cause TdP [7]. Anthracyclines prolong the QT interval by inducing effects on ion channels [8]. QT interval changes have also been linked to tamoxifen and its metabolites (4-hydroxytamoxifen), most likely as a result of the human ether-à-go-go-related gene (HERG) potassium channel blockade [9]. Both QT interval prolongation and TdP have been linked to platinum compounds, particularly oxaliplatin. By extending the sodium (Na^+^) channel opening, oxaliplatin can enhance Na^+^ influx and impact cardiac repolarization [10]. A 49-year-old African American man suffering from cutaneous T-cell lymphoma (CTCL) was reported to have developed TdP after receiving the histone deacetylase (HDAC) inhibitor vorinostat for his treatment [11]. Many tyrosine kinase inhibitors, such as crizotinib, ponatinib, nilotinib, cediranib, vandetanib, lapatinib, sunitinib, etc., are associated with QT interval prolongation [12].

Previous studies have attempted to estimate the use of QT-interval-extending drugs and the occurrence of QT prolongation due to DDIs in patients with cancer. The current study systematically reviews the possibility of DDIs in patients with cancer and the associated risk factors, and provides a quantitative estimate of their pooled prevalence.

## Review

Methodology

Study Protocol

The meta-analysis of observational studies in epidemiology (MOOSE) was employed to perform this review and meta-analysis [13]. The protocol was prospectively registered in the International Prospective Register of Systematic Reviews (PROSPERO) vide reference ID number: CRD42023409522.

Purpose

To estimate the prevalence of therapeutic DDIs causing QT prolongation in patients with cancer. Hence, our P - Population, I - Intervention, C - Comparison, O - Outcome, or PICO elements were P: Cancer patients; I: DDIs causing QT Prolongation; C: not applicable; and O: Potential DDIs.

Inclusion and Exclusion Criteria

The published articles included were observational studies with prospective, retrospective, and cross-sectional study designs reporting the prevalence of DDIs causing QT prolongation in patients diagnosed with cancer who were on chemotherapy. There was no time limit specified. Articles not published in English and those without sufficient data for analysis were excluded.

Data Extraction

The authors developed a standard data extraction sheet in Microsoft Excel (Microsoft Corp., Redmond, WA, US). The data significant to the study, such as the design used, the study setting, target population, total number of patients with cancer, total number of patients with potential QT-interval prolonging DDIs, author details, month and year of publication, and a comprehensive list of drug classes that may potentially cause DDIs were extracted. The desired outcome, such as the percentage of QT-prolonging DDIs and the associated risk factors was also extracted.

Quality Assessment 

The quality of the articles was assessed using the 12-point criteria adopted from a prior study, Nabovati et al., 2014 [14]. The points assessed included the study's objectives, data collection methodology, study setting, inclusion/exclusion criteria, the definition of a DDI and its categories, the DDI categories considered, and reference to a DDI. The sampling population and sample size calculation, potential or actual DDIs evaluated, quality control, and the study limitations listed were also evaluated. The quality assessment was based on the criterion scores of 0 or 1, with total scores ranging from 0 to 12 (scores 0 to 6 indicated poor quality, 7 to 9 indicated moderate quality, and 10 to 12 indicated high quality) (Table 1). 

**Table 1:** Quality assessment of the included studies

Studies	Total score	Quality
Agnihotri et al. (2024) [15]	8	Moderate
Balk et al. (2017) [16]	9	Moderate
Fort et al. (2022) [17]	5	Poor
Hussaarts et al. (2019) [18]	6	Poor
Khan et al. (2017) [19]	8	Moderate
Kim et al. (2019) [20]	9	Moderate
Ramasubbu et al. (2021) [21]	8	Moderate

Outcome measurements

The primary goal was to determine the collective estimate of the DDIs leading to prolongation of the QT interval in patients with cancer. The secondary goal was to determine potential risk factors for the DDIs that cause this QT prolongation.

Data analysis

The pooled estimate of prevalence, i.e., the prevalence of DDIs causing QT prolongation, and subgroup analysis were done by the meta package in R software (R Development Core Team, Vienna, Austria). In this review, the random-effects model was taken into consideration to estimate the clinical heterogeneity among studies. 

Search strategy

**Figure 1:**
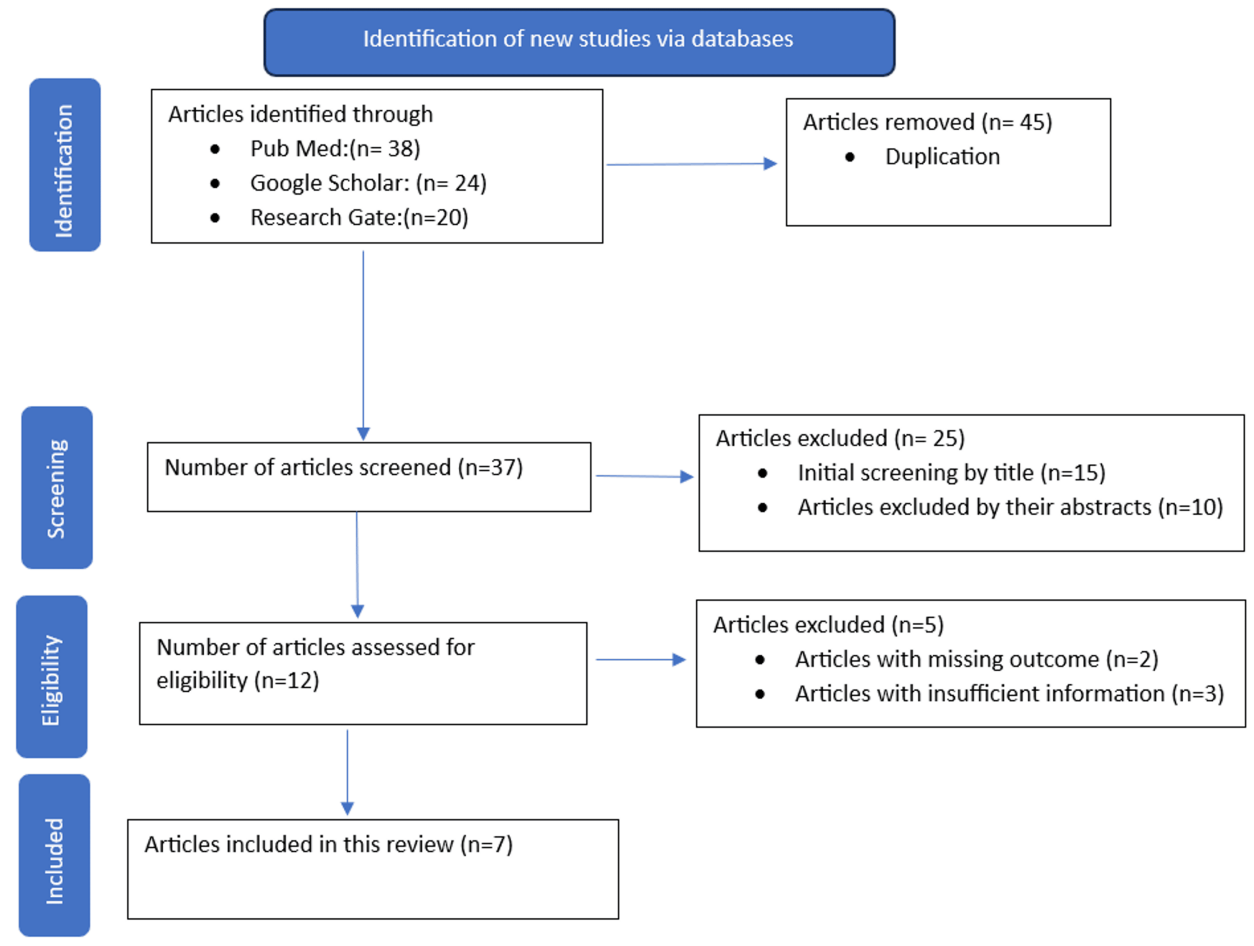
PRISMA flow diagram of the study screening and selection PRISMA: Preferred Reporting Items for Systematic Reviews and Meta-Analysis [22]

A thorough literature search was performed using databases such as PubMed, Google Scholar, and Research Gate with carefully chosen Medical Subject Headings (MeSH) terms (Appendix). There were 82 articles identified through this search. After the removal of duplicates, 37 unique articles were subjected to an initial screening, out of which 25 were rejected as they were deemed irrelevant to the current study. Five manuscripts were eliminated because of insufficient data or missing outcome variables. The final outcome yielded seven articles for our assessment and analysis (Figure 1).

General characteristics of the included studies

Seven unique, relevant, and full-text articles were included in the current study [15-21]. Their important characteristics are presented in Table 2. The study designs of the included articles were cross-sectional, prospective, and retrospectively observational. The year of publication ranged from 2017 to 2024. The studies included patients with cancer treated in out-patient and in-patient settings. Six different evidence-based medicine tools were used to detect potential DDIs in these studies.

**Table 2:** General characteristics of the studies included in the systematic review and meta-analysis OPD, out-patient department; IPD, in-patient department; CS, cross-sectional; OS, Observational study; NR, not reported *potential or clinically-relevant

Author (Year)	Setting	Design	Methods of DDI detection	Types of DDIs* causing the QT prolongation assessed	Quality of the study included	Specific drugs used	Measures taken for the DDIs causing QT prolongation
Agnihotri et al. (2024) [15]	OPD	CS	Drugs.com, Epocrates, Medscape	Potential	Moderate	Capecitabine, Oxaliplatin, Bortezomib, Bendamustine, Domperidone, Ondansetron, Olanzapine, Tramadol	NR
Balk et al. (2017) [16]	OPD	Prospective OS	Micromedex solutions, Dutch drug database	Potential	Moderate	Ciprofloxacin, Methotrexate, Cotrimoxazole, Vincristine, Itraconazole, Aprepitant	ECG monitoring was advised to the treating oncologist
Fort et al. (2022) [17]	OPD & IPD	Prospective OS	Micromedex	Potential	Poor	Sunitinib, Osimertinib, Cabozantinib, Citalopram, Quetiapine, Tramadol	NR
Hussaarts et al. (2019) [18]	OPD & IPD	Retrospective OS	-	Potential and clinically relevant	Poor	Tamoxifen, Selective Serotonin Reuptake Inhibitors (SSRIs)	Baseline and follow up ECG was done
Khan et al. (2017) [19]	OPD & IPD	CS	Micromedex	Potential	Moderate	Ciprofloxacin, Ondansteron, Prochlorperazine, Metronidazole, Dolosetron, Nilotinib	NR
Kim et al. (2019) [20]	OPD & IPD	Retrospective OS	Lexicomp, Micromedex	Potential	Moderate	Tamoxifen, Clarithromycin, Nilotinib, Imatinib, Domperidone	NR
Ramasubbu et al. (2021) [21]	OPD & IPD	CS	Drugs.com, Epocrates, Medscape	Potential	Moderate	Oxaliplatin, Ondansetron, Tramadol, Epirubicin, Fluconazole, Hydrochlorthiazide, Pantoprazole	NR

Prevalence of DDIs causing QT prolongation

The data on DDIs causing QT prolongation are presented in Table 3.

**Table 3:** Prevalence of DDIs causing QT prolongation in the articles evaluated DDI, drug-drug interaction

Study	Total no. of patients	No. of patients with DDIs causing QT prolongation	Prevalence of DDIs causing QT prolongation (%)
Agnihotri et al. (2024) [15]	1331	1295	97.2
Balk et al. (2017) [16]	73	2	2.73
Fort et al. (2022) [17]	171	37	21.63
Hussaarts et al. (2019) [18]	100	1	1
Khan et al. (2017) [19]	555	121	21.80
Kim et al. (2019) [20]	5657	2075	36.68
Ramasubbu et al. (2021) [21]	126	27	21.42

**Figure 2:**
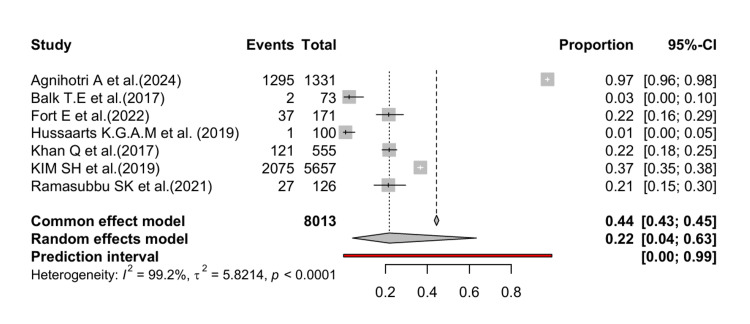
Forest plot depicting the pooled prevalence of DDIs causing QT prolongation in patients with cancer Citations for the included studies [15-21]; DDI, drug-drug interaction

The pooled prevalence of DDIs causing QT prolongation in patients with cancer included in the seven studies was estimated to be 22% with a 95% CI between 4% and 63% (Figure 2). The heterogeneity across the studies was estimated to be high (I² = 99%, p<0.01).

Subgroup analysis

**Figure 3:**
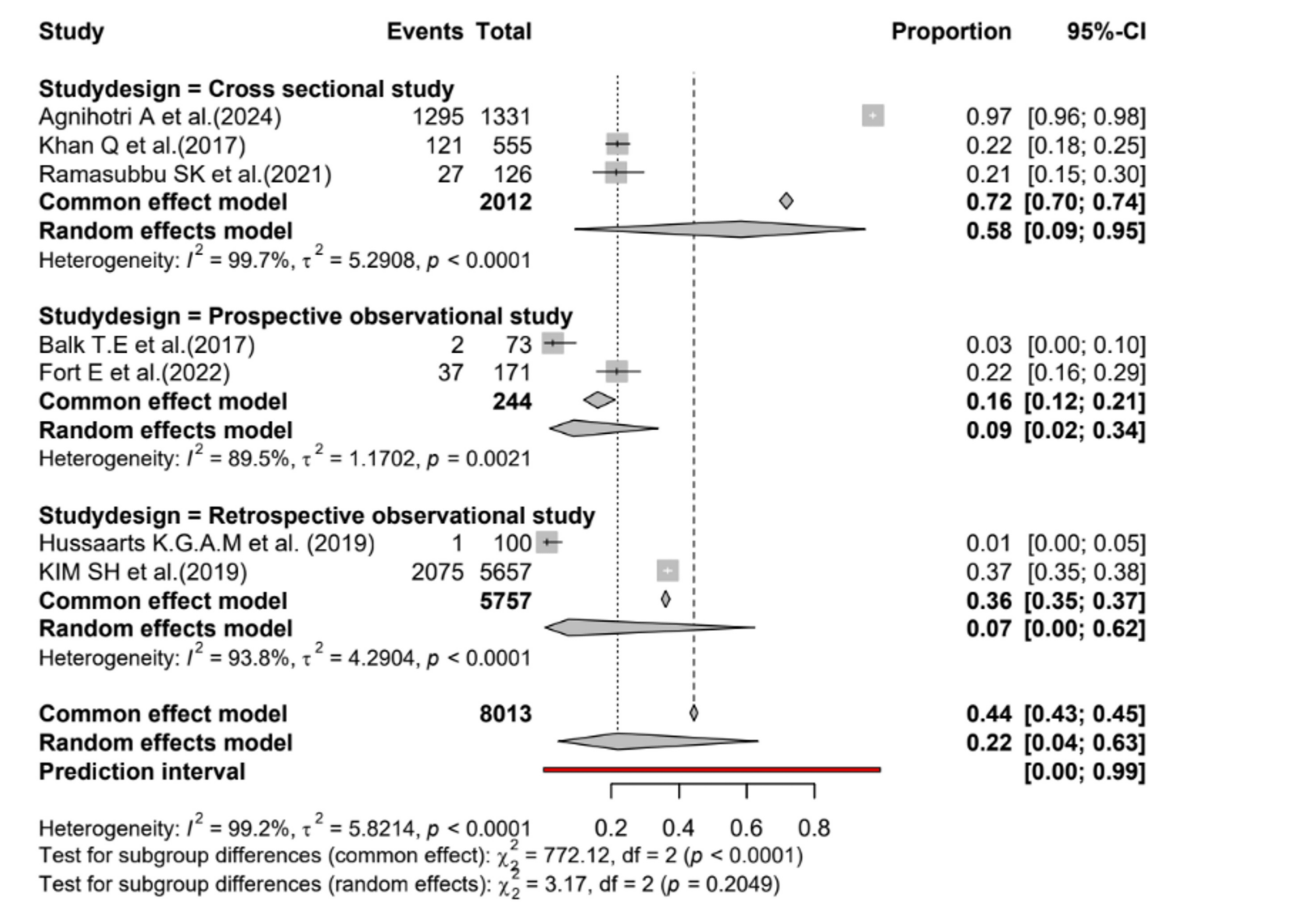
Forest plot comparing the study designs of the studies included in the analysis Citations for included studies [15-21]

A subgroup analysis performed to compare the study designs of the included studies found that there was no difference between the study design and the occurrence of DDIs causing QT prolongation in patients with cancer (Figure 3).

Risk factors associated with the DDIs causing QT prolongation

Factors such as polypharmacy, comorbidities, use of supportive care therapy, and advanced age increased the risk of DDIs causing QT prolongation in patients with cancer (Table 4).

**Table 4:** Associated risk factors for DDIs causing QT prolongation DDI, drug-drug interaction

Factors that increase the risk of DDIs causing QT prolongation	Description
Polypharmacy	Increase in the number and concomitant use of many drugs
Comorbid diseases	Presence of comorbid diseases like diabetes may elevate the risk
Use of supportive care drugs	Use of multiple drugs for supportive care along with cancer chemotherapy can predispose the patients
Age	Elderly patients were found to be more predisposed

Discussion

This systematic review and meta-analysis on the therapeutic DDIs causing QT prolongation in patients with cancer studied the prevalence of such DDIs and their associated risk factors. Data from 8013 patients were included in our analysis to uncover the pooled estimate of the primary outcome. The overall prevalence of DDIs causing QT prolongation in patients with cancer in our study was 22% (4%-63%; 95% CI).

Moghaddas et al. (2021) evaluated therapeutic DDIs in hospitalized patients with cancer and reported that 80.8% of them were exposed to DDIs, which is higher than our study [23]. Another study by Girre et al. (2019) reported that 30.4% of elderly patients with cancer were at risk of DDIs, which is also slightly higher than the present study [24]. These differences could be explained by the various classification schemes used. Studies have shown that healthcare providers may become confused about how to handle the DDIs due to their inconsistent classifications and the information provided by different sources that detail DDIs.

In one of the evaluated studies, the potential DDI was extracted and presented to a committee of experts. After the consensus, if the DDI required intervention, the treating oncologist was informed and advised on how to manage it [16]. In another study conducted on patients with breast cancer treated with tamoxifen and selective serotonin reuptake inhibitor (SSRIs), an ECG was performed to measure the QT interval at baseline and during follow-up [18]. Agnihotri et al. (2024) [15] estimated that 97.2% of the patients were at risk of potential DDIs causing QT prolongation. This finding is in contrast to a study by Hussaarts et al. (2019) [18], where the prevalence was only 1%. This difference in findings may be attributed to the difference in the sensitivity of the software used to detect the DDIs. No DDI database software offers 100% sensitivity or specificity when evaluating them. Notably, there are discrepancies in the grading of severity in this type of software, which poses a challenge to the treating oncologist when they encounter potential DDIs. These inconsistent results could also be caused by differences in the patients' clinical profiles and the prescribing patterns of physicians. Decisions to enforce preventive strategies to combat DDIs causing QT prolongation should not be solely based on potential DDIs causing QT prolongation, as the significant difference in prevalence rates indicates that they are poor predictors of harm caused to the patient.

Polypharmacy can potentiate the risk of DDIs in patients with cancer. Prescribing combinations of anticancer drugs along with drugs for supportive care and concurrent use of other QT-prolonging drugs can heighten the risk of QT prolongation. Studies in elderly patients with cancer show that the prevalence rate of polypharmacy ranges from 2% to 80%, depending upon the population studied and the definition of polypharmacy [25]. A case report by Cerdà et al. showed that a patient on chemotherapy was prescribed a combination of methadone, haloperidol, and fluoxetine. Subsequently, the patient developed cardiac adverse events, presented with chest pain and bradycardia, and was diagnosed with reversible QT prolongation. It shows that polypharmacy can lead to QT prolongation in such patients [26]. Physicians who prescribe anticancer and supportive care medications to patients with cancer should carefully consider the risk factors for QT prolongation.

The presence of comorbid illness also increases the risk of QT prolongation in patients with cancer. Electrolyte imbalances and the therapeutic use of diuretics are two additional risk factors that increase the risk of TdP [21]. Prolonged proton pump inhibitor use can result in hypomagnesemia and its major complications, such as QT prolongation and potentially fatal ventricular arrhythmias [27]. Similarly, prolonged QT interval and hypokalemia are adverse effects attributed to 5-hydroxytryptamine type 3 receptor (5HT-3) antagonists such as ondansetron, which is a commonly used anti-emetic. Additionally, one of the modifiable risk factors for drug-induced TdP is hypokalemia [28]. This study will provide physicians with valuable information on the risks of DDIs causing QT prolongation, an area where data is currently limited.

Recommendations

The following measures can be adopted by the clinician to prevent QT prolongation in patients with cancer. First, address the electrolyte abnormalities by supplementing and maintaining potassium and magnesium levels during chemotherapy. Second, discontinue the use of diuretics as much as possible. Third, identify concomitant drugs that can increase the QT interval. Clinicians should discontinue the offending drug or use an alternative drug that has no impact on the QT interval. Fourth, conduct a risk-benefit analysis to determine the use of an alternative antineoplastic agent. Fifth, before beginning treatment, obtain a baseline ECG to identify any patients who may be predisposed to a prolonged QT interval [29]. Finally, examine the potential DDIs of prescribed medications using a variety of online DDI checking tools and make the necessary adjustments. Physicians need to inform patients and make them aware of the potential risks associated with self-medication as well as the likely hazards of DDIs [21].

Future direction

There is currently no clear guideline for assessing DDIs associated with multiple drugs that cause QT prolongation. This topic should be the focus of future research.

Limitations

Only one study in this meta-analysis had ECG data pertaining to QT prolongation. There may be inconsistencies in the software used to detect the DDIs.

## Conclusions

The prevalence of potential DDIs causing QT prolongation was estimated to be 22% in patients with cancer undergoing treatment. The associated risk factors were old age, polypharmacy, use of drugs for supportive care, and comorbid conditions. Geriatric patients with cancer are at a high risk of developing DDIs with QT prolongation due to polypharmacy. We recommend incorporating QT prolongation and the risk of TdP into patient monitoring plans in clinical practice. It is imperative to adopt precautionary measures to forestall QT prolongation in elderly patients with cancer. The treating physician can identify possible DDIs and make appropriate modifications to the treatment plan by using the online DDI checking software. To optimize drug therapy in patients with cancer, an integrative and algorithmic approach involving general practitioners, cardiologists, oncologists, and clinical pharmacologists/pharmacists is necessary.

## Appendices

**Table 5:** Data sources and literature search strategy

A thorough literature search was performed using databases such as PubMed, Embase, and Research Gate with carefully chosen words, namely “Prevalence”, “QT-prolongation”, “DDIs”, “associated factors”, “cancer”, and “oncology”. AND/OR Boolean operators were used for narrowing down the search in the identification of the relevant articles. The key words used in PubMed were ("epidemiology"[MeSH Subheading] OR "epidemiology"[All Fields] OR "prevalence"[All Fields] OR "prevalence"[MeSH Terms] OR "prevalance"[All Fields] OR "prevalences"[All Fields] OR "prevalence s"[All Fields] OR "prevalent"[All Fields] OR "prevalently"[All Fields] OR "prevalents"[All Fields]) AND ("QT"[All Fields] AND ("interval"[All Fields] OR "intervals"[All Fields])) AND ("drug interactions"[MeSH Terms] OR ("drug"[All Fields] AND "interactions"[All Fields]) OR "drug interactions"[All Fields] OR ("drug"[All Fields] AND "interaction"[All Fields]) OR "drug drug interaction"[All Fields]) AND (("cancer s"[All Fields] OR "cancerated"[All Fields] OR "canceration"[All Fields] OR "cancerization"[All Fields] OR "cancerized"[All Fields] OR "cancerous"[All Fields] OR "neoplasms"[MeSH Terms] OR "neoplasms"[All Fields] OR "cancer"[All Fields] OR "cancers"[All Fields]) AND ("patient s"[All Fields] OR "patients"[MeSH Terms] OR "patients"[All Fields] OR "patient"[All Fields] OR "patients s"[All Fields])). All research articles published till ‘June 2024’ and which met the inclusion criteria were taken into account.
